# Evaluation of macular blood flow after intermittent intravenous infusion of high-dose corticosteroids (pulse therapy) in patients with thyroid-associated orbitopathy (TAO) using angio-OCT

**DOI:** 10.1007/s00417-021-05336-4

**Published:** 2021-09-01

**Authors:** Chiara Del Noce, Matilde Roda, Lorenzo Ferro Desideri, Carlo E. Traverso, Aldo Vagge

**Affiliations:** 1 Department of Neuroscience, Rehabilitation, Ophthalmology, Genetics, Maternal and Child Health (DiNOGMI), Eye Clinic of Genoa, University of Genova, Viale Benedetto XV. 5, 16132 Genova, Italy; 2 IRCCS Ospedale Policlinico San Martino, 16132 Genova, Italy; 3 Ophthalmology Unit, S.OrsoIa-Malpighi University Hospital, University of Bologna, Bologna, Italy

**Keywords:** Pulse therapy, Thyroid-associated orbitopathy, OCTA

## Abstract

**Purpose:**

The aim of this study is to evaluate the changes in macular blood flow index (BFI) in patients with moderate to severe thyroid-associated orbitopathy (TAO) before and after pulse therapy and their relationship with clinical features and disease activity using angio-OCT technology.

**Methods:**

We analyzed twenty-four eyes. Every patient underwent a complete eye examination and angio-OCT analysis (OCT Topcon ImageNet 6; DRI OCT Triton, Topcon Corporation) before (T0) and two months (T2) after pulse therapy. We analyzed macular vascular blood flow in four angiographic levels: superficial plexus (SP), deep plexus (DP), external retina (ER), and choriocapillaris (CC). We used the clinical activity score (CAS) score to define TAO as moderate or severe.

**Results:**

Macular BFI significantly increased at T2 in the DP, ER, and CC (*p* < 0.01). CAS score (5.8 ± 0.8 vs. 3.9 ± 0.9, *p* < 0.01) and Hertel exophthalmometry values (22.6 ± 2.3 mm vs. 21.2 ± 2,5 mm, *p* < 0.01) improved for all patients at T2 compared T0. Mean IOP increased from 13.3 ± 2.8 mmHg to 14.3 ± 2.1 mmHg (*p* < 0.01). No correlation was found between CAS score and macular BFI in all the analyzed levels.

**Conclusions:**

Pulse therapy treatment can change macular BFI. In particular, two months alter pulse therapy, all the patients show an increase in macular vascular blood flow in each angiographic level. According to our results, angio-OCT analysis of the macular BFI may be a useful tool in the follow-up of TAO patients after pulse therapy.

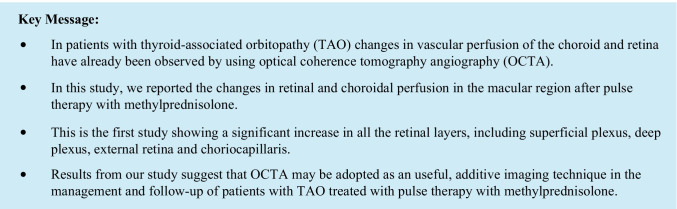

## Introduction

Graves’ disease (GD) is an autoimmune disorder characterized by the excess production of thyroid hormones due to the overstimulation of the thyroid gland by thyrotropin (TSH)-receptor autoantibodies (TRAbs). GD is the most frequent cause of hyperthyroidism, with a prevalence of ~ 0.5% in the general population, with a female-to-male ratio of ~ 3:1 [1–3].

Thyroid-associated orbitopathy is an autoimmune disorder related to GD. It occurs in up to 50% of individuals with GD. However, it may also occur without current or prior hyperthyroidism, or in people who are hypothyroid due to chronic autoimmune Hashimoto’s thyroiditis [4].

Clinical manifestations of active thyroid-associated orbitopathy (TAO) can vary and may include conjunctival chemosis and injection, lid swelling, lid retraction, proptosis, strabismus, exposure keratopathy, and optic [5]. Common symptoms may include eye pain, excessive lacrimation, diplopia, photophobia, and blurry vision [6]. Clinical activity score (CAS) has been extremely used in assessing the activity level of TAO [7].

Active TAO is typically described as mild, moderate-to-severe, or sight-threatening according to EUGOGO guidelines [8].

For active and mild disease, ocular lubricants, sunglasses, and prisms are used for supportive management, for moderate-to-severe disease immunosuppressive therapy strategies are used, while in sight-threatening disease, surgical intervention is often needed [9].

Glucocorticoids are used as a method of medical decompression due to their anti-inflammatory and immunosuppressive properties in patients with severe TAO to prevent disease progression [10]. Pulse therapy is based on intermittent intravenous (IV) infusion of corticosteroids. The standard dosage is the infusion of methylprednisolone 500 mg IV per week for 6 weeks followed by 250 mg IV per week to taper off. Methylprednisolone should be used with caution in patients with diabetes mellitus, hypertension and liver disease. It is absolutely contraindicated in patients with fungal and bacterial infections [11].

The aim of the present study was to analyze the effects of IV high-dose methylprednisolone therapy in patients with TAO and the relationship between changes in macular vascular blood flow investigated by angio-OCT technology.

## Materials and methods

This study was a retrospective study. All participants gave their informed consent and the study followed the principles of the Declaration of Helsinki. The study was conducted at the University Eye Clinic of Genoa, Department of Neuroscience, Rehabilitation, Ophthalmology, Genetics, Maternal and Child Health (DINOGMI), IRCCS Ospedale Policlinico S. Martino, Genova, Italy. It involved patients affected by moderate to severe TAO disease who underwent pulse therapy. According to EUGOGO classification we included patient with lid retraction ≥ 2 mm, moderate to severe soft tissue involvement, exophthalmos ≥ 3 mm above normal and constant or inconstant diplopia. Exclusion criteria were mild or sight-threatening TAO, previous orbital surgery or radiotherapy, myopia greater than − 3 diopters, IOP > 18 mm Hg and other diseases that affect retinal and choroidal circulation such as glaucoma, uveitis, retinal and choroidal diseases and carotid artery stenosis.

All subjects diagnosed with TAO were evaluated at baseline (T0) and 2 months after the last infusion of pulse therapy (T1) by the same physician.

At T0 and T1, all the patients underwent a complete eye examination including best-corrected visual acuity, biomicroscopic examination, intraocular pressure (IOP) measurement, Hertel exophthalmometry, and indirect ophthalmoscopy.

TAO activity was assessed through the clinical activity score (CAS). CAS is composed of a list of seven inflammatory orbital symptoms representing pain, redness, and swelling. A CAS of three points or more indicate the patient is in an active phase of the disease [12].

After pupillary dilation, all patients included in the study were evaluated with angio-OCT scans using OCT Topcon ImageNet 6 (DRI OCT Triton, Topcon Corporation).

OCT angiography scans were obtained from an area of 4.5 × 4.5 mm^2^ centered on the fovea.

We analyzed macular BFI in four angiographic levels: superficial plexus (SP), deep plexus (DP), external retina (ER) and choriocapillaris (CC). Macular BFI.

was calculated as the percentage of black and white pixels present in the scan assuming that this percentage represents an indirect measure of vascular flow.

### Statistical analysis

The data are reported as mean values ± standard deviation and include data on the right eyes. Parameters of the two groups were compared using Student *t*-test for continuous variables and chi-squared test for categorical variables. Macular vascular blood flow index before and after pulse therapy was compared using the independent sample *t*-test. The values with *p* < 0.05 were considered statistically significant, and all *p* values were based on two tailed tests.

## Results

Twenty-four right eyes of patients with moderate-to-severe TAO (8 men and 16 women), aged 25–75 years (mean 54.8 ± 15.2), were studied.

**Fig. 1:**
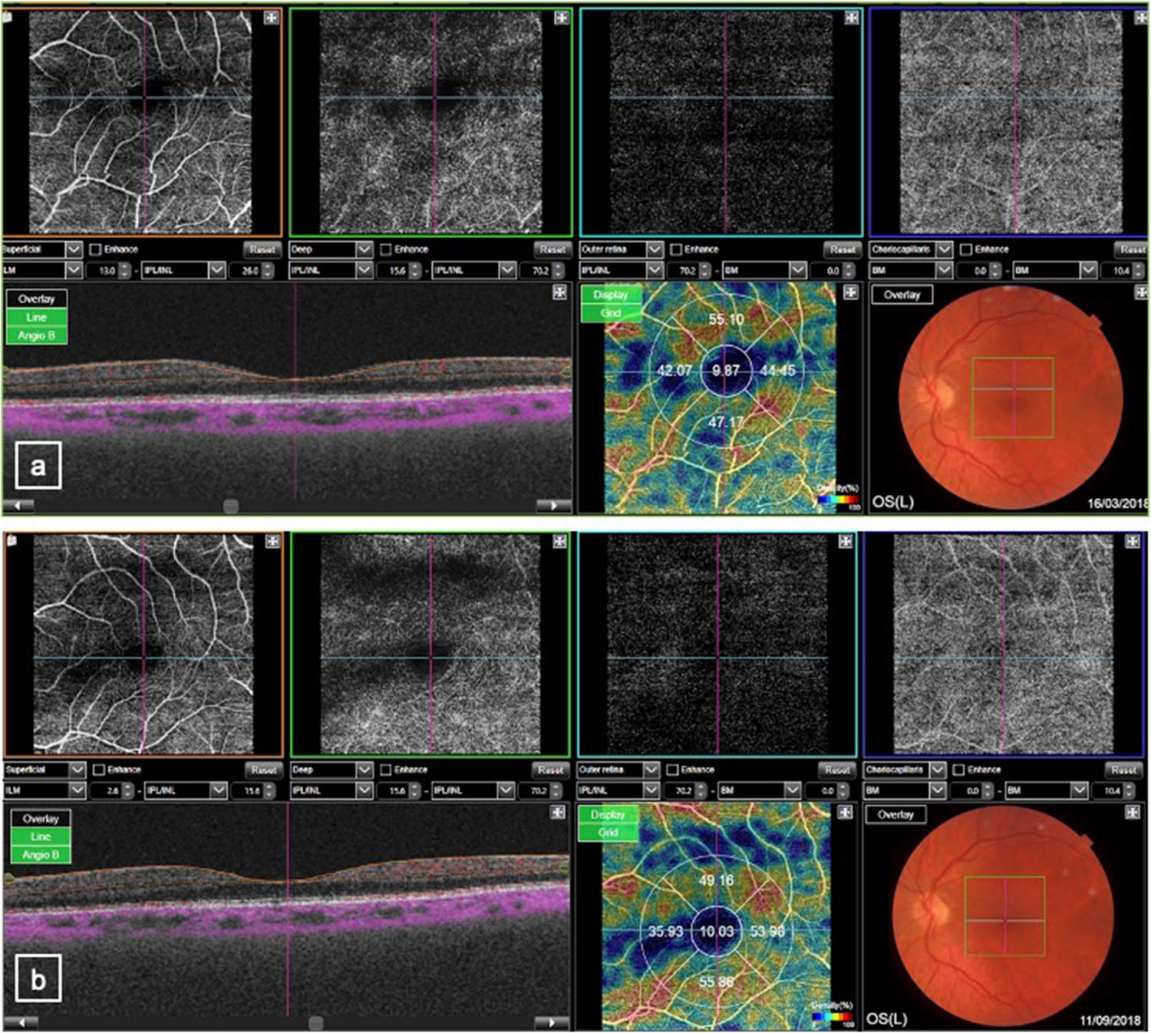
**a** Optical coherence tomography angiography (OCTA) images before starting pulse therapy. Above from left to the right: superficial capillary plexus, deep capillary plexus, external retina, choriocapillaris. Below from left to the right: radial acquisition, density map, and fundus photography. **b** Same OCTA images after 2 months from pulse therapy; in the density map, it is evident the increase of the vascular density in the deep capillary plexus after the pulse therapy

Macular BFI at T0 was lower in every angiographic level compared to macular BFI at T1. Mean values for DP BFI, ER BFI and CC BFI after pulse therapy (43.6 ± 2.2, 47.9 ± 1.6, 48.9 ± 1.7, respectively) were significantly higher (*p* < 0.01) than the pretreatment values while no statistically significant values were found for SP BFI (*p* values > 0.05) (Table 1) (Fig. 1).

**Table 1:** Macular blood flow index (BFI) values measured by angio-OCT at four different retinal levels before pulse therapy (T0) and 2 months after pulse therapy (T1)

	T0	T1
SP BFI (% ± SD)	38.5 ± 3.7	38.9 ± 2.5
DP BFI (% ± SD)	42.5 ± 2.2	43.6 ± 2.2*
ER BFI (% ± SD)	45.8 ± 1.6	47.9 ± 1.6*
CC BFI (% ± SD)	47.3 ± 2.1	48.9 ± 1.7*

*SP BFI* superficial plexus blood flow index, *DP BFI* deep plexus blood flow index, *ER BFI* external retina blood flow index, *CC BFI* choriocapillaris blood flow index;

*%* percentage, *SD* standard deviation

^*^p < 0.01 T0 vs. T1

Visual acuity remained stable for twenty patients. In four patients there was an increase in visual acuity at T1 (0.65 ± 0.05) compared to T0 (0.35 ± 0.09, *p* < 0.05). No correlation was found between visual acuity and macular BFI in every level.

Fifteen patients complained diplopia before treatment. After treatment, diplopia disappeared for three patients and remained stable for the remaining twelve. Sixteen patients had corneal involvement before treatment which resolved at the end of therapy for fourteen patients.

Mean (± SD) CAS score evaluation was changed from 5.8 ± 0.8 to 3.9 ± 0.9 by pulse therapy (*p* < 0.01).

No correlation was found between CAS score and macular BFI in every level.

Mean (± SD) proptosis values were significantly decreased after pulse therapy (from 22.6 ± 2.3 mm to 21.2 ± 2.5 mm, *p* < 0.01).

Mean IOP measurements at T1 (14.3 ± 2.1 mmHg) were significantly higher than those at T0 (13.3 ± 2.8 mmHg, *p* < 0.01).

CAS score, proptosis values and IOP measurements are summarized in Table 2.

**Table 2:** Clinical activity score (CAS), Hertel exophthalmometry measurements and intraocular pressure (IOP) values before pulse therapy (T0) and 2 months after pulse therapy (T1)

	T0	T1
CAS (*n* ± SD)	5,8 ± 0,8	3,9 ± 0,9*
Hertel exophthalmometry (mm ± SD)	22,6 ± 2,3	21,2 ± 2,5*
IOP (mmHg ± SD)	13.3 ± 2.8	14.3 ± 2.1*

*CAS* clinical activity score, *n* number, *SD* standard deviation, *IOP* intraocular pressure

^*^p < 0.01 T0 vs. T1

No serious adverse events occurred in any patients. Six patients (25%) reported mild hypertension and palpitation after pulse therapy.

## Discussion

GD-related ophthalmopathy is a chronic, autoimmune disease that requires a long period to recover. Numerous therapies are available to control the progression of orbital pathology, avoid damage to the cornea and the optic nerve and prevent ocular motility disorders. Although the most effective of the drugs used for immune suppression seem to be steroids, high oral dosage is necessary for a long time, and causes serious adverse effects [13]. IV high-dose steroid pulse therapy is reported to be more effective and adverse effects are less [14].

Moreover, IV steroid therapy is more effective and better tolerated by patients than oral administration [15]. However, several adverse events associated with pulse therapy have been documented including severe hepatic insufficiency [16]. Side effects were in relation to dose, treatment regimen, age and patient’s comorbidities [17]. Patients should be screened for recent hepatitis, cardiovascular disease, hypertension, liver dysfunction, diabetes and glaucoma. Therefore, careful patient selection is mandatory. It is recommended to maintain a dose range between 6 and 8 g and to visit the patient monthly during treatment [18]. It has also been reported that tighter monitoring is advised in patients older than 53 years as they have a higher risk of hepatic failure at high doses [19]. However, steroid treatment remains the first choice therapy for patients with moderate to severe TAO [20].

Several other studies have been conducted to investigate retinal vascular changes in patients with TAO using OCT angiography. Lanchu et al. evaluated retinal and choroidal variation in TAO patients by angio-OCT. They observed that patients with TAO had significant changes in RNFL thickness, choroidal thickness and superficial retinal vessels. Patients with inactive TAO had greater retinal vascular density than healthy controls [21].

In the study by Tehrani et al. the average peripapillary vessel density was significantly lower in patients with active TAO compare to the other groups [22]. Similarly, Dave et al. found that in patients with active TAO peripapillary and macular area vascularization were reduced in patients with active TAO compared to healthy controls [23].

Another study analyzed choriocapillaris vascularization using angio-OCT and found that in patients with TAO choriocapillaris vascular flow was markedly reduced compared to healthy controls of the same age and sex [24]. Ocular hemodynamic changes in patients with TAO have also been studied by the group of Lei et al. On the contrary, they found an increased superficial retinal layer and deep retinal layer microvascular density in the macular area in patients with TAO [25].

This study observed changes in macular vascularity in patients with active TAO treated with pulse therapy. In particular, it has been studied that macular vascularization increased after pulse therapy in all retinal levels: superficial plexus, deep plexus, external retina and choriocapillaris. These results can be explained based on the fact that in active TAO there is an increase of the musculature and of the intraorbital soft tissues that determine an obstacle to the normal retinal vascularization. After therapy with IV methylprednisolone, the anti-inflammatory power of cortisone determines a resolution of muscle edema and intraorbital soft tissues involvement, allowing to resolve vascular flow obstruction and increasing retinal vascular index.

We recognize that this study has limitations as it is retrospective and has a small cohort.

Further studies are necessary to analyzed pulse therapy effects on retinal vascularity and to establish its role in appropriate long-term follow-up in a larger sample of patients.

In conclusion this study demonstrated a change in macular vascular blood flow in patients with active TAO who undergoes pulse therapy treatment. In particular, two months after pulse therapy, all the patients showed an increase in macular vascular blood flow in each angiographic level. According to our results, angio-OCT values of macular vascular blood flow may be added in the evaluation of patients who undergo pulse therapy.
